# Dominant and opponent relations in cortical function: An EEG study of exam performance and stress

**DOI:** 10.3934/Neuroscience.2018.1.32

**Published:** 2017-12-30

**Authors:** Lucia P. Pavlova, Dmitrii N. Berlov, Andres Kurismaa

**Affiliations:** 1 Department of Higher Nervous Activity and Psychophysiology, Faculty of Biology, St. Petersburg State University, St.-Petersburg, Russia; 2 Department of Anatomy and Physiology of Humans and Animals, Herzen State Pedagogical University of Russia, St.-Petersburg, Russia; 3 International Research Center of the Functional Materials and Devices of Optoelectronics and Electronics, ITMO University, Saint Petersburg, Russia; 4 Department of History and Philosophy of Science, Faculty of Science, Charles University in Prague, Czech Republic

**Keywords:** cortical activity, dominant principle, electroencephalogram, functional asymmetry, individual variability, opponent processes

## Abstract

This paper analyzes the opponent dynamics of human motivational and affective processes, as conceptualized by RS Solomon, from the position of AA Ukhtomsky's neurophysiological principle of the dominant and its applications in the field of human electroencephalographic analysis. As an experimental model, we investigate the dynamics of cortical activity in students submitting university final course oral examinations in naturalistic settings, and show that successful performance in these settings depends on the presence of specific types of cortical activation patterns, involving high indices of left-hemispheric and frontal cortical dominance, whereas the lack thereof predicts poor performance on the task, and seems to be associated with difficulties in the executive regulation of cognitive (intellectual) and motivational processes in these highly demanding and stressful conditions. Based on such knowledge, improved educational and therapeutic interventions can be suggested which take into account individual variability in the neurocognitive mechanisms underlying adaptation to motivationally and intellectually challenging, stressful tasks, such as oral university exams. Some implications of this research for opponent-process theory and its closer integration into current neuroscience research on acquired motivations are discussed.

## 1. Introduction

In the current paper, we focus on two basic, interrelated principles of systemic regulation of brain functions—the opponent process theory by R.S. Solomon [1]–[3], and A.A. Ukhtomsky's principle of the dominant [4],[5], and apply them to electroencephalographic (EEG) analysis of human performance at university oral exams in naturalistic conditions [6]. By drawing on the experimental results of this pilot study, we demonstrate that successful adaptation of students to the requirements of an oral examination depends on the presence of individual types of cortical activation patterns (CAPs), involving high indices of left-hemispheric and frontal cortical dominance, whereas the lack thereof reliably predicts low achievement on the task, and seems to be associated with difficulties in the executive regulation of cognitive (intellectual) and acquired motivational processes in these highly challenging and stressful conditions. Findings from these studies seem to support several key tenets of Solomon's opponent process theory of motivation dynamics [3], and may help to analyze its so far relatively poorly understood neurophysiological mechanisms in the light of the dominant principle [4],[5]. In particular, the widely prevalent, if not universal functional principle of coupled opposed dynamics (COD) of cortical activity, as revealed in the principle of the dominant, can be of fundamental importance for elucidating how functional cerebral systems with mutually exclusive and opposed effects interact in time, leading to both adaptive or maladaptive behavioral and cognitive responses. We introduce functional measures of COD, such as the coupled inversion of anterio-posterior (fronto-occipital) and bilateral (inter-hemispheric) activation gradients, to analyze these responses, and show how their dynamics change in different task conditions and cognitive states in a manner consistent with the opponent process theory.

Methodologically, analyzing the neurophysiological dynamics of motivational reactions in ecological settings may require specific approaches, and this has been rarely attempted in exam conditions. While numerous works are devoted to the role of emotions, stress and anxiety in the learning process [7]–[9], including the exam situation, virtually all such studies are limited to pre-examination and post-examination analysis [10]–[15], without affecting the exam itself, particularly with regard to measuring the brain's bioelectric activity in the course of the exam interaction and presumable peak stress experience. The current line of studies sought to validate the applicability of dynamic EEG analysis in these settings [6],[16],[17]. It may therefore represent particular interest for analysing not only the electrophysiological correlates of opponent processes, as understood by Solomon, but also for considering their so far little explored social and interpersonal aspects in relevant natural settings.

As will be shown, based on such knowledge, the individual variability of dominant and opponent processes can be analyzed, and improved pedagogical and therapeutic interventions suggested which take into account marked individual differences in the neural and cognitive mechanisms underlying adaptation to motivationally and intellectually challenging tasks, such as the oral exam. These aspects will be more extensively addressed in the discussion, after the concepts of the dominant and opponent processes have been introduced (section 2), and relevant empirical materials presented (section 3). Theoretically, the integrative approach developed here [6],[16],[17] corresponds to the widely recognized need for systemic frameworks and methodologies in the fields of behavioral and human neuroscience [18],[19], and in the analysis of EEG [20],[21], in particular.

## 2. Dominant and opponent processes

In the fields of neuroscience and psychophysiology, both the theory of opponent processes, as well as the principle of the dominant stand out by their systemic, heuristic predictions and specific applications in an unusually wide range of topics. Thus, Solomon and Corbit [1] proposed a general model of opponent processes to explain an apparently widespread mechanism securing the dynamic homeostasis of intense, contrastive emotional and motivational states [3]. The authors gathered evidence from physiology and psychology for a general model explaining how intense hedonic experiences can automatically induce in the nervous system a biphasic, compensatory motivational or affective process of opposite hedonic valence, before a return to stable affective baseline state occurs in the subject. However, the neurophysiological underpinnings of this dynamic homeostatic phenomenon have remained relatively elusive and little studied, in comparison to its behavioral and psychological effects.

Recently, some of the related methodical and methodological challenges have been discussed by Comer et al. [22]. In particular, the authors propose that the functional cerebral systems theory of A.R. Luria [23] may still provide “unsurpassed explanatory value and testability” in promoting the systemic-dynamic exploration of functional processes within the nervous system [22], including the relevant homeostatic and compensatory effects. Indeed, such aspects have remained largely underappreciated, and challenge current attempts to integrate opponent processes into mainstream neuroscience research, according to their view [22].

Here, we suggest that besides the works of A.R. Luria, valuable insights for the study of dynamic functional systems can be obtained from a historically and methodologically closely related tradition, namely A.A. Ukhtomsky's study on the dominant [4],[5]. The fundamental basis informing this line of work concerns the unity of opposed functional processes in the brain—excitation and inhibition—as tonic neurophysiological states, and their reciprocal induction in cortical and neuronal excitability [24]–[26]. In particular, this approach may help to understand how intense work-load on any functional system—of immediate hedonic valence or not—can evoke its auto-inhibition and resultant “super-compensatory” effects, before a more stable baseline of excitability is restored or modified in the brain. In the present paper, we are limited to discussing this phenomenon in its cortical physiological aspects.^1^

The dominant approach allows to highlight how the opponent temporal dynamics of motivations and emotions may depend on the non-equilibrium properties of the cortical biopotential field as a whole. This field can be characterized by transitions in the *foci of maximal activation* (FMA), and by the localization of the accompanying coupled, collaterally inhibited areas in the surrounding cortical tissue. These two contrastive neurophysiological responses represent a pattern of *coupled opposed dynamics* (COD) in the cortex that seems to be of wide, perhaps universal relevance for interpreting neurophysiological coordination dynamics and mechanisms [6],[17],[27] (section 3).

In particular, this approach to opponent processes may allow to better understand the mechanisms governing dynamic changes in hemispheric dominance [22],[28], as well as to demonstrate how shifts in inter-hemispheric and prefrontal dominance relate to changes in the motivational and higher cognitive processes of subjects as they adapt to diverse task conditions and cognitive work load [6],[16],[27]. Below, we show evidence for the hypothesis that opponent motivational processes may be directly related to changes in hemispheric and prefrontal dominance indices. While this hypothesis has been proposed and is supported by other experimental paradigms and evidence [22],[28], the current approach allows to extend and generalize these findings by applying a novel experimental and methodological framework for their neurophysiologically rigorous and ecologically valid investigation—albeit in a small-scale pilot study.

It can be noted that respective materials raise also general theoretical problems, as they highlight that shifts in motivational states are most probably not limited to the sphere of “hedonic” processes or specific subcortical regions in the brain, but seem to involve widely distributed functional cerebral systems, including cortical ones associated with higher psychological processes and executive functions in humans. Although direct EEG evidence on opponent effects is so far limited, a recent study by Kline et al. [28] has obtained relevant results in this regard and should be shortly highlighted.

The authors revealed the role of prefrontal cortical regions in the opponent-type regulation of emotional experience, and showed how the organization of this experience depends on the dynamics of hemispheric functional asymmetry. In particular, it was shown that fear reactions evoked in a group of participants (in response to aversive pictures of human faces) are accompanied by increased relative right prefrontal activation, whereas the predominance of left prefrontal regions inhibits the same negative reaction and may, in well-coping subjects, respectively show enhanced and super-compensatory activity after the initial fear response. The authors interpret this increased leftward activation as a contrastive after-reaction necessary for suppressing, on an opponent process basis, the mainly right-hemispheric aversive response [28]. Although not obtained in an exam setting, these results seem to confirm the view that opponent affective processes, as conceived by Solomon, are closely associated with a corresponding contrastive dynamics in frontal lobe activity. A replication of this hypothesis in other experimental paradigms would be highly desirable, nevertheless, to demonstrate the pervasiveness of such opponent regulations and their possible functional contexts. This could also lead to a better understanding of the intra- and inter-individual variability which such opponent effects may have, their task-specificity, as well as association with other neural systems.

Close to the present focus, an early study by Craig and Siegel [29] has addressed the principle of opponent regulation in the exam situation. The authors investigated habituation to test-anxiety in college students and obtained evidence supporting Solomon's theory. In particular, by administering mood adjective checklists to students for self-rating just before and immediately after taking a final course exam, the authors quite expectably found a reliable decrease in dysphoria—but more significantly, also an increase in euphoria subsequent to the stressful test event, consistent with the opponent process model [29]. The important implication of the latter is the prediction of not simply attenuating apprehension, but also a surge in elation upon completing the exam. However, this study did not employ any physiological measures, and together with other related studies on exams [10]–[12],[30], would clearly benefit from an integrated psychophysiological approach, allowing to analyze the neural substrates and mechanisms directly involved in the exam situation and interactions [16],[17]. Likewise, most research on emotional and stress reactions has so far investigated EEG and peripheral signals separately [31], although their fusion and joint assessment may improve the robustness of both lines of findings [31], as our own data in the exam setting also suggests.

As shown below, our research on the higher cortical regulation of cognitive and motivational processes are in direct agreement with the above results [28],[29], and may help to generalize and extend these findings on opponent-type regulation to more complex types of motivational and cognitive responses—such as involved in real-time exam situations. Before turning to the empirical findings obtained in this framework, we will briefly describe some methodological specifics of this line of studies based on A.A. Ukhtomsky's principle of the dominant. Further integration of this approach and findings with opponent-type processes is presented in the discussion.

## 3. Theoretical preliminaries: The dominant and human EEG

It's passing first to consider A.A. Ukhtomsky's pioneering insights on the functional role of EEG rhythms. Based on the concept of “operative rest” or calm (cf. [32],[25]), his views were among the first to clarify the controversial issue of the quasi-periodic alpha-rhythm (8–10 Hz) and its significance in human brain activity. Ukhtomsky proceeded from the experimental fact that in humans, the resting state is dominated by coherent, low-frequency alpha-waves of high amplitude. Peripheral stimuli from sense organs are known to disturb this “resting-state oscillation” and to give rise to higher-frequency activation (beta rhythms > 12 Hz) in the cortical projections, further enhanced by the subject's endogenous attentional and emotional arousal. These facts led Ukhtomsky to conclude that it would be *incorrect to see coordination as being generally based on the synchronization of neuronal activity alone* [4]—more often than not, *it depends on a parallel increase in the desynchronization of neural networks* [4],[6]. This constitutes a general principle of *coupled opposed dynamics* (COD) in brain function, as clarified below.

Elucidating the role and mechanisms of alpha-rhythm desynchronization continues to be an active area of research, where various general and more specific hypotheses have been offered to account for its functions. Jensen et al. [33] have framed an influential view on the gating and filtering properties of the cortical alpha, which through targeted suppression (“pulsed inhibition”) of higher-frequency rhythms, particularly gamma oscillations (30–70 Hz), is assumed to have an active inhibitory role in shaping functional cortical architecture. Closely compatible interpretations have been recently proposed by Klimesch [34], who suggests an active inhibitory function for alpha activity in controlling attentional and conscious access to stored memory and knowledge; for this access to occur, information from competing sources must be temporarily excluded (suppressed). In more formal terms stemming from information theory, alpha desynchronization can be related to information richness in the brain, necessary for the encoding and retrieval of memory and other cognitive processes [35]; on the other hand, the degree of synchrony in neural firing patterns is inversely related to their information carrying capacity [35]. Indeed, hypersynchronized cortical activity in the alpha range has been associated with complete blockage of intracortical communication, leading to the breakdown in sensory processing and loss of consciousness [36]. Important studies, closely related to our own, have also been carried out in the framework of coupled event-related desynchornization/synchronization (ERD/ERS) by Pfurtscheller and colleagues, suggesting that cortical activation (reflected in ERD) may be more focused and concentrated when surrounded by fields of antagonistic inhibitory synchronization (ERS), particularly within the alpha band [37]–[39].

Thus, modern studies seem to offer numerous confirmations regarding the dominant concept and its application in the field of EEG study. At the same time, some methodological differences regarding the principles of EEG analysis should be noted. This concerns above all the problem of dynamic features of neural signals, specifically the non-stationary (discontinuous, segmentary) and stochastic properties they exhibit. While knowledge of such features has been available for a long time (and forms the basis of our work [40]), they have typically been ignored in current and classical frameworks of EEG interpretation due to methodical and theoretical premises [20],[21]. On the other hand, while this may simplify signal analysis, neglecting such dynamic features has also lead to significant difficulties in constructing global models of the EEG phenomenon, and in relating it to problems of cognition and consciousness [20],[21]. Thus, novel methodologies sensitive to the underlining quasi-stationary nature of the EEG signal are clearly necessary [21]. One of the earliest such frameworks has been developed in collaboration with one of the authors (L.P.) [17],[40] on the basis of the dominant principle. Below, some of its key premises and methods are briefly outlined.

The principle of dominant introduces into cognitive science a factor rarely considered in other frameworks—the factor of non-equilibrium as an invariant principle in all neurocognitive phenomena. In its most general form, Ukhtomsky characterized dominant states as consisting of two coupled and inverse processes—a leading “focus” or excitatory link, and systemic propagation of inhibition over the remaining elements of the system. This divergent pattern constitutes a universal mechanism of coordination in his view, and the means by which superfluous degrees of freedom are eliminated in neural systems. In this context, dominance is not so much a theory or hypothesis, but an obvious feature of functional cerebral systems in his view. However, it can offer powerful heuristics for studying brain activity when constrained by specific models and analytic methods, and may prove to be its highly universal organizational feature.

It is thus instrumental to define an adequate model for dominant states and the associated non-equilibrium dynamics in brain networks. The dominance model outlined below presents methods for multi-parametric and multi-channel analysis of such functional dynamics according to coherence and synchrony parameters [6],[40]. Accordingly, the activation gradients (AG) characterizing functional asymmetry indices along anterio-posterior (AP) and bilateral (LR) interhemispheric cortical zones define the structure of cortical activation patterns (CAPs), and their “non-equilibrium” (functional asymmetry). In our previous works, we elaborated optimal statistical quantitative measures for characterizing functional shifts in the brain's dominant CAP states, defined by the momentary activation gradients between α- and higher frequency rhythms [40],[17] (Appendix 1).

In this model, a dominant CAP state is reflected in two coupled inverse shifts in regional biorhythm indices characterizing cortical areas: (1) a focus of maximal activation (FMA), with amplified β-rhythms in a given region and attenuated α-oscillations (down to their complete disappearance in that area); and (2) a state of coupled inhibition in the surrounding cortical regions, as reflected in the simultaneous appearance of amplified α- rhythm [6],[17] (Appendix 1).

Additionally, an activation coefficient K_C/O_ can be determined by the relation of latent reaction periods (LRP) after closing and opening the eyes—with LRP for closed eyes (LRP–CE) reflecting excitation inertia, and LRP for opened eyes (LRP–OE) reflecting inhibition inertia, or the inertial properties of inhibitory cortical states (Appendix 2).

## 4. Experimental studies on exam performance

Oral exams present one of the most intense forms of human mental activity, combining both intellectual, emotional, and stress-regulatory components in a highly dynamic social setting [6]. Examining their individual variability and neurocognitive structure may therefore present unique insights into the mechanisms of opponent processes in naturalistic conditions.

### 4.1. Methods and materials

Our studies were carried out in an experimental EEG recording facility in collaboration with Dr. N. Volkind from Krasnoyarsk Pedagogical Institute, with whom we conducted university term examinations on the subject “physiology of higher nervous activity” on volunteering student participants from St.-Petersburg State University's Psychology Faculty. To ensure high performance criteria, students' examination grades were recorded on exam sheets and reflected in their official study records.

Experimental conditions: In a group of 20 students (18 y.o., male, all right-handed), EEG was recorded continuously from 8 to 10 symmetrical anterior and posterior cortical sites (using the device “Biofizpribor”, 0.3–100 Hz bandwidth), simultaneously with electrocardiogram (ECG) data [6],[17]. Electrode montage is specified in Appendix 3. On the eve of the exams, a test experiment was carried out on each participant to ensure habituation to exam settings and to the Eyes Closed/Eyes Open (EC/EO) test (Appendix 2). Each experimental session lasted for no less than 1.5 hours in a row, during daytime, under normal daylight conditions. EEG recordings were made as the subjects were seated in a comfortable chair, in a specially screened room (3 × 3 m^2^) shielded from external noise. After installing the electrodes, the FAM test (Feeling, Activity, Mood) [41],[42] was administered to students, who thereafter were left alone for 15 minutes to rest and prepare before starting the exam. After completing the exam, students were left to rest for 20 minutes, before being again administered a FAM test by the experimenter. Furthermore, prior to the experiment we tested subjects by the Hand [43], personal orientation inventory (POI), and Eysenck personality questionnaire (EPQ) psychological tests.

Students' EEG and ECG were recorded continuously throughout 5 stages of the exam: I stage—students await for the examiner, corresponding to a state of operative rest (15 min); II stage—the examiner enters the room, students receive tickets (topics), read them in the examiner's presence, the examiner leaves; III stage—students prepare independently an answer to the ticket (20 min); IV stage—students are orally examined on the ticket and on additional questions, are notified of their grade (20 min); V stage—period of post-exam rest, the examiner has left (20 min). Throughout the whole exam, short EC/EO tests were administered every 2–3 min.

It should be stressed that we did not assess FAM scores by averaging results across the participants, but distinguished between 2 experimental subgroups by their grades—a high-achieving group (A), who passed for “excellent”, and a low-achieving group (B), who either failed the exam or passed it poorly. This strategy was used to reveal adequate correlations between CAP types and given sets of activity. While selecting students to be included in either group by their grade, we strove to maintain their homogeneity also by other indices, above all by high achievement motivation, which was present in all subjects. (In group A, all 5 students had “excellent” academic records exclusively in all subjects, and had all graduated with honors from highschool. In group B, students with high achievement motivation and generally good knowledge of the subject were chosen, but who failed to demonstrate this knowledge in the specific settings of an oral exam, both in the current study and during prior oral exams). These inclusion criteria were applied meticulously, particularly given the small sample size of the study.

Ethical conditions: The study was conducted on unpaid volunteers. Experimental procedures of study, including its ethical and medical aspects, were reviewed and approved by an expert committee at the A.A. Ukhtomsky Physiological Research Institute at St.-Petersburg State University. Participation in the study involved written consent from students and Deans of the Psychology and the Biology Faculties of the University.

### 4.2. Results

**Figure 1.**
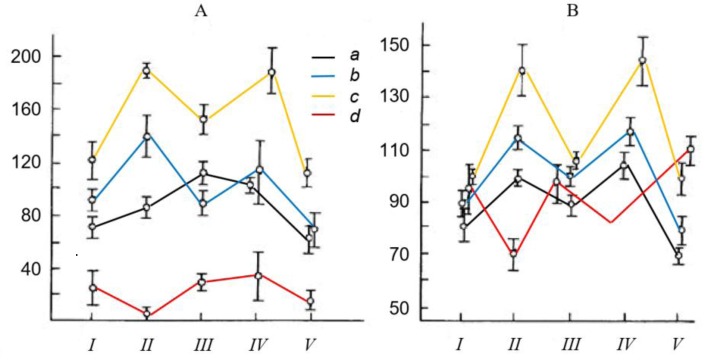
Shifts in general activation of cerebral cortex (A) and pulse rate (B) in consecutive stages (I–V) of oral examination in four variously graded groups (5 subjects in each group). *Ordinate*: A—average measures of general cortical activation (ΣK_C/O_, in conditional units), B—pulse rate (bpm); *a, b, c, d*—grades received: excellent *(a)*, good *(b)*, average *(c)*, poor *(d)*, respectively. Abscissa—exam stages: *I*—waiting; *II*—drawing a ticket; *III*—preparing the answer; *VI*—exam response; *V*—after-effects.

Most significant shifts in the level of cortical activation (by the coefficient K_C/O_) (Appendix 2) and vegetative nervous activity (pulse rate) were observed in stages II and IV of the exam—while drawing the ticket and answering it, respectively. Signs of examination stress were particularly pronounced in highly anxious, poorly answering students (Figure 1).

**Figure 2.**
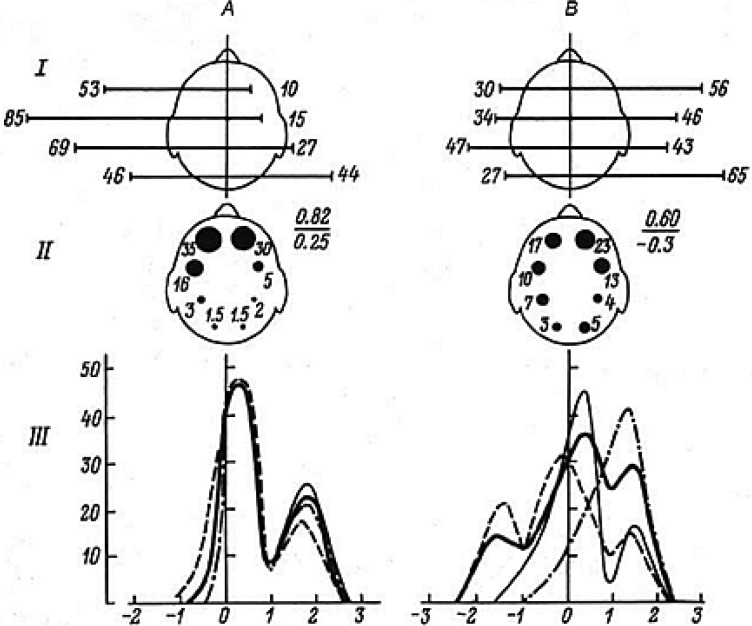
CAP types and mental work productivity at university exams (average data in 2 groups, 5 people in each). A—students with “excellent“ results, generally high-achieving subjects; B—students with average or poor results, generally lower-achieving subjects. I—relative activation (by K_C/O_) of left and right symmetrical cortical zones (%). II—regional activation indices (on hemispheric projections; conditional units); numbers on the right: numerator—anterio-posterior non-equilibrium (K_A/P_), denominator—bilateral asymmetry index (K_L/R_). III—variational distribution of K_C/O_ values on logarithmic scale (on abscissa), number of variants (*n* = 400) for each value (on ordinate). One curve corresponds to one subject. Values within K_C/O_ > 0 reflect activation, values within K_C/O_ < 0 reflect marked inhibition, deactivation. For methods, cf Appendix 2.

Significant individual differences in the indices of general cortical activation by K_C/O_ (Figure 1A) and pulse rate (Figure 1B) can be seen in relation to success rate at the exam. During all stages of the exam, students receiving excellent and good grades (groups a and b) showed intermediate values for these indices, in comparison to students receiving average and poor grades (groups c and d). Thus, less successful responders where characterized either by an excessive degree of cortical activation and pulse rate (group c), or an insufficient value of these functional indices (group d), in comparison to the high-achiving groups. The reliability of this data is increased by the identical conditions in which all examinees were tested, and the highly significant differences in functional brain states of high- and low-achieving participants (Figure 2).

Additionally, consideration of background EEG signals at the exam complements materials obtained by the EC/EO test, and allows to reveal symptoms of stress as well as mental fatigue in students. Most pronounced general cortical activation, determined by the coefficient of relative β- and α rhythm power (K_β/α_), was observed in stages II and IV of the exam, and was accompanied by most significant increases in pulse rate (by 1.5–2 times).

Below, we analyze the CAP types in two groups of students with most divergent results at the exam, respectively receiving “excellent” or “poor” (insufficient) grades. Both groups included five subjects, who were tested during the exam (by EC/EO test) no less than 60 times each. The high significance of obtained differences is reflected in the variational curves obtained from large sample sizes (*n* = 400) of the EC/EO test in the two student groups (Figure 2).

In the “excellent”—graded group of students (Figure 2A), stable FMA was observed in left frontal areas by the general activation level as well as by the percentage of prevalent left-sided activity on the background of high anterio-posterior (fronto-occipital) activation gradients (AGs). At the same time, the almost complete superposition of K_C/O_ variational curve values, revealing the presence of a distinct FMA in left frontal areas, testifies to a largely identical functional brain state in all five high-achieving subjects. Double-peaking variational curves reveal a distinct FMA in left frontal areas on the background of significantly reduced activation range in the subdominant brain regions, with a non-significant transition rate in the deactivated areas (by K_C/O_ < 0; cf. Appendix 2). This allows to speak of a correspondence between the identified CAP type and requirements posed by the given class of verbal-logical tasks.

Among students receiving average and poor grades (group B), no distinct and stable FMA was found on the background of predominantly right-hemispheric activity (Figure 2B). In this group, diverse types of individual variational curves are seen, as well as a wider range of functional states (FSs). There is significant variation in regions with increased (K_C/O_ >0), as well as decreased activation, the latter reflecting an inhibited cortical FS (K_C/O_ <0) (Appendix 2). Reduced mental working capacity is accompanied by predominant activity in right prefrontal areas, on the background of significantly diminished anterio-posterior AG.

Comparing exam stages IV and V—the oral response and post-exam rest (after the examiner has left)—leads to the suggestion that opponent-type functional states, as described by R. Solomon [1]–[3], characterize also cognitive performance during exams. This is reflected in the shifting activation indices for the left and right hemispheres, and accompanying changes in mood and feeling by FAM test (discussed below). During exam stage V, an interesting paradoxical reaction can be seen in the brain activity of highly anxious subjects: a state of defensive cortical inhibition characterizing the response period typically changes, after the examiner has left, to a relatively normal state with FMA in frontal cortical regions; at the same time, speech functions recover that had been suppressed in the student during the response period in the examiner's presence.

**Figure 3.**
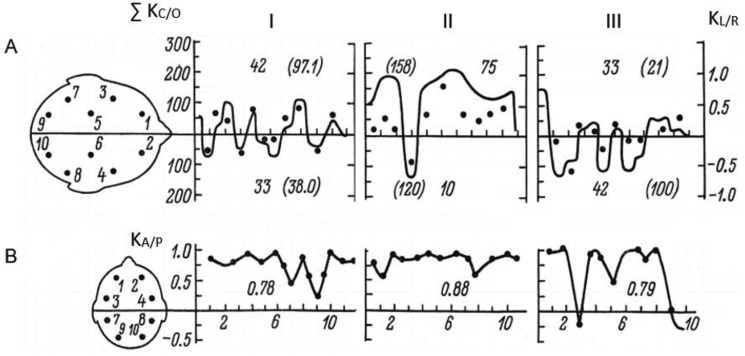
Examples of individual EEG dynamics during three examination stages in an excellently graded student (R.). A—diagram of the summed activation index of left and right cortical hemispheres (∑ K_C/O_, left ordinate). Deviations above midline—functional predominance of left-hemispheric activity, below midline—right-hemispheric predominance; numbers above and below curves: in brackets—summed general cortical activation (∑ K_C/O_), without brackets—relative activation predominance (%); isolated dots—values of inter-hemispheric asymmetry (K_L/R,_ right ordinate). B—magnitude of anterio-posterior non-equilibrium (K_A/P_, ordinate); numbers below curves—averaged activation value; on abscissa—number of EC/EO trials (dots). I—before exam start; II—while answering the ticket; III—after exam termination (examiner has left). Differences in the scale for summed cortical activation in hemispheres (0–300) and their functional asymmetry (−1 to 1) are due to respective equations (measurement units are conditional) (Appendices 1 and 2).

**Figure 4.**
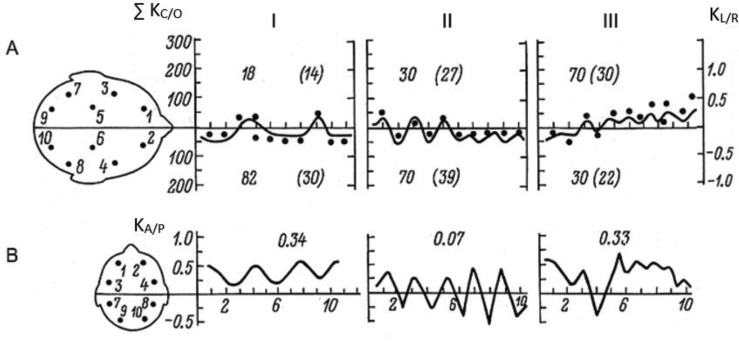
Examples of individual EEG dynamics during 3 examination stages in a poorly graded student (G.). Same designations as in Figure 3.

Below, a detailed comparison of functional brain states during key stages of the exam, I, IV and V, are shown for two most highly contrastive students (Figures 3 and 4; Table 1). The students belong to different grade groups (Figure 2): Student R. was the best among high-achievers, while student G. the poorest performer in the weaker group. Data on intra-individual and comparative time-series variation can be particularly informative given the non-Gaussian distribution of obtained within- and between group measures (Figure 2), as well as considering the marked variabilty of individual EEG indices across various stages of the exam (Figures 3 and 4).

**Table 1.** Individual EEG, heart-rate and emotion indices of a highly-graded subject (R.) and a poorly graded subject (G.).

Examination stage	**Student R.**			**Student G.**		
	**I**	**IV**	**V**	**I**	**IV**	**V**
Left hemisphere activation index	97	158	21	14	27	30
Left hemisphere dominance	42%	90%	33%	18%	30%	70%
Right hemisphere activation index	38	20	100	30	39	22
Right hemisphere dominance	33%	10%	42%	82%	70%	70%
K_L/R_	0.4	0.7	−0.6	−0.3	−0.2	0.5
K_A/P_	0.7	0.8	0.7	−0.3	−0.07	0.3
Heart-rate (bpm)	73	85	69	105	125	95
FAMai	6.0		4.5	2.2		5.3

Hemispheric activation indices are in conditional units (Appendix 2). I, IV, V—stage of experiment; K_L/R_—bilateral asymmetry index; K_A/P_—anterio-posterior asymmetry index; FAMai—FAM-test averaged scale index. Subject R. received an excellent evaluation, subject G.—poor evaluation.

Significant differences in the dynamics of cortical functional state can be seen in the representative highly-graded subject R. and poorly graded subject G. As seen on Figures 3–4, this difference is manifest already before exam onset, during the waiting stage (operative rest). This is reflected in the general level of cortical activation, which is significantly higher in student R. on the background of left-hemispheric dominance (shown as dots on Figures 3A, 4A), and the significantly higher (0.78) and more stable predominance of frontal cortical regions (Figures 3B, 4B). In student G., right-hemispheric dominance can be seen on the background of significantly reduced cortical activation and appearance on the EEG of slow hyper-synchronous delta-waves, reflecting cortical defensive inhibition already prior to exam onset. At the same time, anterio-posterior functional asymmetry is markedly diminished (0.34) due to deactivation of frontal brain regions.

These differences between students R. and G. increase during the response stage (II). In the high-achieving student R., left-hemispheric dominance is strongly amplified (with rising general activation), and the stability of frontal activity is increased. In student G., right-hemispheric dominance is retained on the background of reduced activation and instable dominance of frontal areas.

However, after exam completion, in both students rapid shifts occur in the opposite direction: in R., there is a transition to right-hemispheric dominance with a sharp drop in general cortical activation and reduced stability of frontal dominance, which can be interpreted as a reduction in neurocognitive work load. In student G., on the other hand, left-hemispheric dominance is quickly increased after the examiner has left, together with increases in inter-hemispheric functional asymmetry and frontal activation, *i.e.* cortical activation is increased.

**Figure 5.**
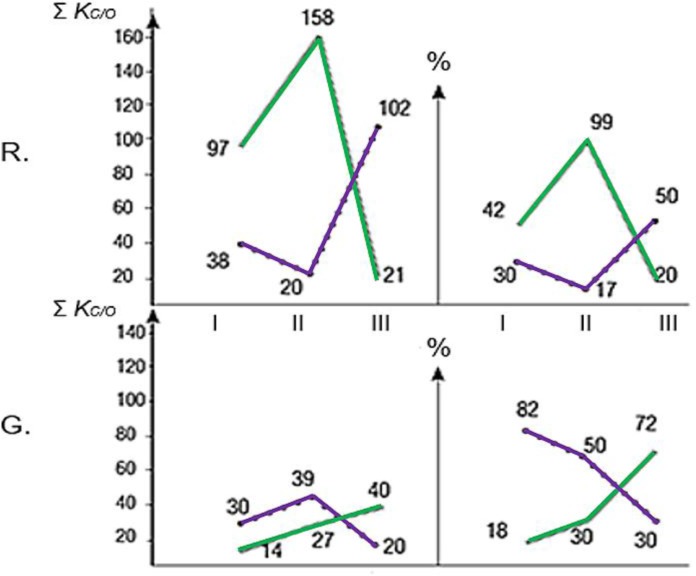
Prevalence of left- and right-hemispheric activation (by K_C/O_) at three stages of the exam. Green—left hemisphere; purple—right hemisphere. Above—student R., excellent response. Below—student G., poor response. Abscissa: I—Initial state; II—response; III—after-effects. Left ordinate—activation sum by K_C/O_ (curves in left columns); right ordinate—relative dominance of LH and RH, percentage (right columns).

On the example of these two students, strongly opposed intra-individual functional brain states can be seen by EEG and pulse measures when comparing stages 4 (response) and 5 (examiner's departure) (cf. Table 1 and Figure 5).

Additionally, before and after the exam, we administered to all students the FAM test [41],[42] on feeling, activity and mood changes (Table 1). The range of functional state (FS) shifts on this test lies on a scale from 1 to 6 points. Normal FS is considered to lie between 5.0–5.5 points; scores below 4 reflect poor FS and mood. In the high-performing group (Figure 2A), the average score prior to the exam was 5.5, and fell to 3.6 post-exam; in the unsuccessful group (Figure 2B) the average pre-exam score was 3.5, and rose to 4.9 after exam termination. In student R., the pre-exam score was 6.0 (highest in group A), but fell to 4.5 post-exam (by mood factor). In student G., the pre-exam score was 2.2 points; however, 20 minutes post-exam the score had risen to 5.3 (by mood factor) (Table 1).

Although these results are preliminary and need careful replication on larger samples, it should be noted that the corresponding changes in neural activity observed across task conditions in each group seem to confirm them. In particular, this concerns the widely reported associations of relative left frontal activation with positive emotions and approach motivation, versus the negative emotions and defensive motivation associated with right-hemispheric frontal functions [28],[44]–[46]. This asymmetry has also been directly observed in the context of examination stress regulation [10]. In this light, let us consider the two students' indices more closely.

Student R., with low anxiety, prevalence of verbal intellect, and analytical cognitive style (by Eysenck EPQ test), obtained an excellent grade. His initial functional state is characterized by left-hemispheric dominance, which increases during the exam response on the background of significant elevation of fronto-occipital AG and some increase in pulse rate. During this period, right-hemispheric activation decreases due to collateral inhibition from left-hemispheric dominants.

During the response, student R. shows positive emotions, apparently takes pleasure in answering the questions posed by the examiner. However, after responding, there is a clear drop in mood according to the FAM test (Table 1). At the same time, a significant reduction in left-hemispheric activation can be observed, together with increased activity and dominance of the right hemisphere, as well as diminished fronto-occipital AG, as shown in Table 1.

We can see from the above data how a clear transition takes place in student R., from a highly active physiological and cognitive state (and positive emotional experience) to an opposite functional state (accompanied by notably declined mood after the examiner has left). This is also reflected in the contrastive changes of EEG indices and pulse rate (Figure 5; Table 1).

Student G. shows high anxiety, has synthetic cognitive style, and prevalence of non-verbal intellect (by Eysenck's EPQ test). The subject knows the material, but has since school-years been afraid of exams. In the initial state, his cortical activity is reduced on the background of right-hemispheric dominance and markedly increased pulse rate (Figure 5; Table 1). Anterio-posterior AG and inter-hemispheric functional asymmetry are reduced. During the response, general cortical activation somewhat increases in the right hemisphere on the background of reduced activation and percentage of left-hemispheric activity, as well as deactivation of frontal regions (reflected in low fronto-occipital AG). However, as the examiner leaves, indices of functional brain state change contrastively—an increase is observed in left-hemispheric activation, together with increasing fronto-occipital AG on the background of collateral inhibition of the right hemisphere (Figure 5). In this case, a transition could be seen from negative emotional experience in the presence of the arousing stimuli (answering the ticket) to the appearance of a contrastive reaction—relief and satisfaction after the stressful situation has ended (as the examiner left).

A variety of methods were used to determine the emotional state of subjects. In addition to the oral response, the FAM test as well as certain behavioral characteristics and anamnesis were used. Student R., who received the excellent grade, had received during his two years of study at St.-Petersburg State University only the highest marks. Student G. did not manage to pass the exam and received a “poor”grade. During the response his speech was inhibited, and he did not seem to understand well the questions he was asked. Significant cortical deactivation was observed, and this coincided with markedly increased pulse rate (105 bpm) even before the exam, as well as during the response (125 bpm). This state can be defined as involving defensive inhibition (“functional pessimum”) in the cerebral cortex on the background of simultaneous cardiac acceleration. It can be noted that this student, as well as others in the given group (Figure 2B), had frequent breakdowns during exams regardless of sufficiently good knowledge in the subjects. In all high-achieving students (Figure 2A), on the other hand, high mental productivity and composure were observed on the background of stable left frontal FMA while answering the ticket.

Apparently, the reason for CAP “dissolution” in stressful conditions is related to excessive stimulation of the cortex by the ascending activating systems. This gives rise to a flow of tonic impulses that are amplified in conditions of novelty and stress, and remain insufficiently regulated by the cortex. According to Luria's [23] and many later studies [47],[48], key functions in the top-down regulation of ascending activating impulses are fulfilled by frontal cortical structures—a view supported also by Kline et al.'s [28], as well as our own findings.

Furthermore, prior to the exam we tested both subjects by the Hand [43] and POI psychological tests. In student R., we found high directiveness (13 points) and high self-actualization (40 points); in student G.—high frustration (88 points), high anxiety (44 points), and low self-actualization (6 points).

From a methodological perspective, it may be revealing to compare the above results with the findings by Dayan et al. [42], who used the FAM test and cardiac activity measures to study examination stress among high-school students enrolled in general educational classes versus differential classes (with more intensive coursework). FAM test scores demonstrated different dynamics of FS change depending on the type of class the students were enrolled in, as seen in Table 2.

**Table 2.** Average FAM-test scores in students of general and differential education programs.

Measurement time	Average FAM scores in general program	Average FAM scores in differential program
Regular day	5.24 ± 0.22	5.09 ± 0.19
Pre-exam	5.10 ± 0.25	4.85 ± 0.17
Post-exam	4.99 ± 0.25	4.95 ± 0.29.

FAM: Feeling, activity mood (test)

Thus, in the differential (intense coursework) class the scores were somewhat lower (4.85) than in the general class, possibly due to a sense of hightened responsibility for exam results. Higher examination stress was found in sympatotonics. However, in this study FAM scores were averaged across all students of a given class, without differentiating between highly and poorly performing subjects, as in our study. This may explain the less significant differences observed in the above summed FAM test results (Table 2). In other words, representative groups were not defined in either class by academic achievement motivation or actual progress, which may explain the differences from our findings.

Importantly, the above findings seem to indicate that processes with an opponent-type organization affect also higher cognitive functions dependent on strong motivational and emotional arousal. Of course, further similar studies including other groups of students and larger sample sizes are necessary to confirm and extend these findings. However, this should be done in representative groups (*e.g.*, highly motivated subjects), such as the reported groups of psychology students (all of whom were motivated to achieve high grades). Even then, regardless of possessing sufficient knowledge, some participants received low average or even poor grades since they were unable to concentrate, maintain composure, and cope with the stresses presented by the examination setting. In high-performing students, stable left-hemispheric dominance and strong fronto-occipital activation gradients helped to cope with the stressful situation. In low-performing students, on the other hand, no distinct left-hemispheric and frontal FMAs were observed, and this resulted in lower grades and stress-resistance in the same objective examination setting.

In sum, the above results reveal marked differences in the CAPs of successful and unsuccessful students at the exam, as reflected in the significantly higher activation of left frontal regions in high-achievers and of right frontal areas in those who failed the exam or passed it poorly. However, it should be stressed that in both groups, the characteristic CAP structure was regularly replaced by a symmetrically opposite one, with the predominant FMA periodically shifting to the right hemisphere in high-achievers, and conversely, to the left hemisphere in low-achievers, depending on particular stages of the exam. These inverse changes were combined with changes in pulse rate and the affective state of subjects, as registered by the FAM questionnaire and judged subjectively by the examiners at the exam interview. Together with available data on the contribution of prefrontal regions to the lateralization of emotions [10],[22],[44]–[46], these results suggest a key role of frontal brain regions' dominance shifts in the task-specific regulation of motivational and emotional states, including their opponent dynamics [28].

Further, the obtained results show not only the relevant role of activational asymmetries in bilateral hemispheric regions, but also in anterior and posterior brain regions, the relative dominance of which must likewise be regulated in accordance with task settings. Increases in left frontal activity in high-achievers were coupled to decreased, highly structured and stereotypical activation of posterior brain regions. On the other hand, the activation of right frontal regions in low-achievers was associated with higher, more generalized and individually varied activity in posterior cortical areas. Thus, the CAPs revealed in high-achievers were found to be relatively uniform in distribution (Figure 2) compared to low-achievers, in whom higher divergence between individual variational curves was observed, reflecting a wider range of distinct cortical functional states (Figure 2). Similar results on the higher variability of EEG indices during the exam period in low-achieving students have been reported by Wiet et al. [14].

## 5. Discussion

The present study has shown that in exam settings, individually specific reorganizations of CAPs can be observed in students, accompanied by corresponding shifts in their motivational-emotional and cognitive processes. In the light of the opponent process model of homeostasis, we find the indications of dynamic “super-compensatory” effects in inter-hemispheric and anterio-posterior interactions to be particularly interesting, as observed in students under high work-load and exam stress conditions (Figures 3, 4). Thus, after periods involving high activation and relative dominance of either hemisphere or prefrontal regions, these functional activation indices are typically not simply downregulated to the baseline, but show a steep decline below it, accompanied by increased activation in the opposite hemisphere, or posterior regions (Figures 3, 4), in comparison to the initial functional state. This type of super-compensatory regulation seems not to be addressed in the classical frameworks of homeostasis, although as revealed by current and earlier related studies [1],[3],[28],[29], it may represent a phenomenon of potentially wide adaptive significance in the self-regulation of excitability in cerebral functional systems [22],[28].^2^ Theoretically, the opponent principle of regulation seems also consistent with current attempts to extend the classical frameworks of neural homeostasis by concepts such as anticipation and allostasis, to emphasize the inherent temporal variability and complexity of homeostatic processes in the brain [49]–[51].

Earlier, Craig et al. [29] investigated students' emotional dynamics during the high exam session, and found them to closely match Solomon's concept of opponent-type regulation. Our research has led to closely comparable findings. However, unlike in any prior studies, our study included integrated physiological and EEG measures, which were analyzed together with emotional and cognitive processes immediately in exam conditions, and while taking into account the various success rate of responders in high- and low-achiving groups. This is most important for distinguishing between the qualitatively different patterns of psychophysiological response expectable in subjects who not only achieve different grades, but who may experience the whole exam situation and challenge differently in terms of the motivational, stress-regulatory, and affective dynamics involved [9],[52]. Indeed, in line with growing appreciation of the positive roles of stress in motivation and performance [53], Strack et al. [9] have recently shown how the stressful period immediately leading up to the exams can be experienced by some students as motivating rather than threatening or emotionally exhausting, indicating they interpret anxiety as facilitative to learning, and are less likely to appraise the exam stressor as a threat. While this ability is positively associated with academic performance, and prevents emotional exhaustion [9], it is also expectable that the opponent effects in such students would be manifestly different from those who experience the exam, or the days leading up to it, as primarily a negative and threatening stressor [9],[52], with adverse health impacts [10],[12].

Thus, although we have underscored the importance of differentiating between participants based on their performance to overcome such difficulties, there are further methodological and technical challenges to be addressed in this line of research. This includes, besides organizational difficulties, the relatively high diversity of motivational and emotional reactions involved in the exam situation, owing both to individual trait differences [30],[54], as well as to individual expectations and experience in taking exams, the degree of preparation [55], and the subjective significance of the academic result [55],[56]. For this reason, we enlisted only highly motivated and well prepared subjects in our study, and assigned them to different groups based on test scores before comparing the physiological data. Even then, besides group-averages, data on within-individual variability can be instrumental for understanding the neurocognitive structures and dynamics underlying successful and unsuccessful responses. In this way, the possible unique characteristics and strategies of responders can be characterized, together with their individual psychophysiological profile and state.

It should be noted here that most neurophysiological studies on opponent processes to date have looked at cases of pathological dysregulation, mainly addictive behavior and its underlying neurobiological circuitry, changes in which show obvious maladaptive dynamics—and probably involve pathological super-compensatory effects as described by the opponent-process model [57]. On the other hand, besides such obviously dysregulatory effects in neural substrates mediating motivational states [57], and other allostatic effects involved in pathology [49], the opponent type regulation seems to also reflect key principles underlying normal adaptation with a positive and adaptive temporal trend. For example, this has been revealed in sports and physical exercise, the accompanying motivational and affective dynamics of which seem to reveal similar biphasic fluctuations, at least under more strenuous and intense exercise leading to increased resilience and stress tolerance (*e.g.*, *via* stress-induced analgesia by endogenous opioids) [58]. Recently, this biphasic dynamics has been associated also with increased frontal asymmetry measures on a possible opponent process basis [59],[60], similarly as we demonstrate here for the exam setting. Together, this may allow to hypothesize a close integration between higher cortical, emotional, and bodily stress-regulatory responses, on the basis of shared or similar opponent effects in the neural circuits mediating them.

Before turning to more general theoretical and methodological implications of our findings, it is therefore appropriate to comment on their potential applied significance. Indeed, the facts obtained in the current study reflect not only theoretical concerns, but also practical interest regarding the functional diagnostics of students' functional state in educational settings, and in particular, prior to stressful tasks such as (oral) exams. This offers the prospect of detecting “risk groups” most prone to the possible adverse health effects of such educational tasks. Given the increasing rates of anxiety and stress among college students found in recent research [61]–[63], and their close relation to depression and other mental health problems [63], these have become particularly urgent requirements today, and are now challenging universities to continually evaluate the mental health of students, as well as to tailor programs of prevention and treatment sensitive to their individual needs and work specifics [61],[63]. Based on the dominant principle and relevant findings, we can suggest several non-invasive measures to increase the resilience of cortical functions and work dominants in easily stressed, highly anxious, and chronically tired students.

(1) In students practicing sports, symptoms of cortical over-excitation or defensive inhibition were generally not observed during exams. This allows to speak of optimal relations between intellectual, emotional, and stress-regulatory components of the exam response in physically trained subjects [6], and is in accord with numerous findings on the neurocognitive benefits of exercise [64],[65], even if its relations to opponent neural dynamics require further study [58],[59]. In particular, defining universal dose-response relations between excercise vigor, motivational and affective opponent effects, and health benefits has remained a difficult and largely elusive task [58]. From the present perspective, this further underscores the need to develop methods sensitive to the individual variability and specificity of such integrated physiological responses. Below, we discuss this question in more detail with respect to EEG analysis.

(2) Development of self-control through neurofeedback [66]–[68]. Our results have shown increased neurofeedback effectiveness if, in each individual, a most “controllable” cortical zone is selected, in which the alpha-rhythm can be most easily amplified by neurofeedback signals through visual, or other feedback channels [69]. Neurofeedback sessions are found to increase the efficiency of mental work and optimize cognitive performance on the background increased left-hemispheric dominance and fronto-occipital activation gradients [6],[46], in accordance with the above reported results.

(3) The stress impact of an exam can be reduced by changing how students are engaged—*e.g.*, by allowing a written reply, additional time for preparing responses, encouraging attitudes by the examiners, etc. In anxious and neurotic subjects this creates conditions for forming a sufficiently stable frontal left FMA and is accompanied by improved quality of the exam response [6],[16]. Furthermore, we have found evidence for possible personal compatibility effects in student-examiner interactions based on the similarity of their resting-state hemispheric dominance patterns [6],[17]. The possible influence of such effects on a student's performance and grading should be taken into account, particularly in low-achieving students most prone to examination stress and anxiety.

Although traditionally, educational problems have been solved in the confines of humanities, the reported findings clearly indicate how a psychophysiological framework may support and enhance educational practices. This is particularly relevant for meeting special educational needs [16]. To best address these requirements, we propose that distinct types of integrative methods and concepts are needed to analyze not only inter-individual and quantitative, but also intra-individual and qualitative physiological measures of adaptation and human performance [70]. With regard to EEG analysis, this requires particular attention to the dynamic features of the EEG signal, such as its non-stationary stochastic properties [40]. While methods ignoring these complex properties have led to important discoveries, such as the functional specificity of individual EEG frequency bands, the initially rapid temporal resolution of the EEG signal is usually lost under such conditions [20],[21], and makes its neurophysiological systemic interpretation more difficult. This limitation may particularly affect most dynamic experimental settings, such as those analyzed above, involving human psycho-social and socio-physiological functioning in exam conditions, or other conditions involving prolonged and conflicting motivational and stress responses.

In line with these requirements, we have presumed here that instead of individual frequency bands or correlational dependences between them, the neurophysiological units of cognitive processes should be sought in the rapidly shifting, discontinuous metastable states of the brain's biopotential field as a whole, characterized by anterio-posterior and inter-hemispheric activation gradients, as well as by global and regional changes in cortical states' inertial (“trace”) properties (Appendices 1, 2). Such dynamic indices are highly variable both intra-and inter-individually, and this in close dependence on task conditions. Such methodological and methodical aspects may be fundamental if neuroscience research results are to be more directly applicable to educational settings and classroom scenarios, as currently called for [71]. Besides questions of methods and modeling, however, also ethical concerns should be further addressed in this line of research [72], including the possibilities of optimal educational and therapeutic interventions, preventive and rehabilitative measures at the individual level [16], as discussed above.

In our view, the framework of the dominant and the theory of opponent processes could provide valuable, mutually reinforcing concepts and models in this regard. These two frameworks are not only closely compatible, but both seem to possess the optimal levels of generality and complexity expected for integrative explanations and models in theoretical neuroscience [18]. Indeed, the necessity for such concepts—both sufficiently generalizable, yet well specifiable due to adequately chosen basic parameters—is becoming increasingly apparent in the field [18], together with some of the risks associated with prematurely formalizing its subject matter by methods drawn directly from other, non-biological disciplines (informatics, physics, etc.) [17],[18],[40],[73]. These methodological considerations have played an important role in designing the current framework of EEG analysis on the basis of the dominant principle [6]. As such, it is hoped the presented materials encourage further research on the neural dynamics mediating opponent processes, and their integration into theoretical and applied human neuroscience.

## Footnotes

1 Currently, the concept of *hormesis* is widely discussed as a general biological model of state-dependent functional effects in physiological systems, particularly in relation to the varying and opposed effects which neurotransmitters and other substances may have at the cellular level, depending on their concentration (dose-response effects) or other conditions of exposure [77]. On the other hand, the conceptual and historical parallels of the hormetic research paradigm with the framework of parabiosis and paranecrosis, going back to Ukhtomsky's teacher N.E. Wedensky's and D.N. Nasonov's works [78],[79], are also recognized [80],[81]. These aspects lay beyond the scope of this paper, but are important to theoretically highlight, particularly given the recent interest in hormetic phenomena in the nervous system [82],[83]. Without addressing such opposed phased functional effects, it may be difficult to develop a low-level neuronal interpretation of opponent processes in relation to particular transmitter systems, as currently sought [84],[85], and thus firmly ground opponent process theory in biology. Interestingly, the school of Wedensky-Ukhtomsky appears to remain the only physiological tradition where principles characterizing homeostatic phenomena at the neuronal level, and the functional state parameters of general, brain-wide dynamics have been investigated from a common perspective [25],[86].

2 Interestinly, the problematic of super-compensation has a long history in Russian stress and sports physiology [87], as well as neurophysiology [25],[88], although this has remained largely unknown in the West [5].
