# Supplementary material for: Dominant and opponent relations in cortical function: An EEG study of exam performance and stress
Source: AIMS Neurosci. 2017 Dec 30;5(1):32–55. doi: 10.3934/Neuroscience.2018.1.32 (PMC7181896; doi:10.3934/Neuroscience.2018.1.32)
Supplement: Supplementary file 1 [file neurosci-05-01-032_s001.docx]

**Appendix**

**Appendix 1**

Work on the basis of the dominant model, as well as facts obtained by various analytical methods have revealed that the EEG correlate of cognitive states does not lay in individual frequency bands of the EEG signal, nor in the presence of correlational dependencies between them in any particular brain regions. Instead, it should be sought in specific metastable states of the cortical “bio-potential field” as a whole over subsequent time-segments [20,21].

Accordingly, we have developed methods for segmenting the EEG signals into intervals of structurally homogenous or uniform activation, separated by rapid transitions in the cortical field potential [17,40]. Similarly to current work on EEG “micro-states“ and quasi-stationary structure [20,21], this approach is still relatively novel, as it avoids averaging EEG signals over extended time periods and broad frequency bands to achieve statistical reliability.

During structurally homogeneous EEG intervals, units of integral brain acitivity are identified as cortical activation patterns (CAPs), which are differentiated by the localization of foci of maximal activation (FMAs). Activation levels in distinct regions can be compared by spectral analysis of β-rhythm (excitation) and α-rhythm (rest) distribution, (K_β/α_), as illustrated on Figures 6 and 7.

The translocation and stability of FMAs in the cerebral cortex, corresponding to periods of structurally homogenous EEG intervals, are shown on multichannel EEG recordings (Figure 7), where the graphic analysis of their spectral characteristics is exemplified. Across diverse activities, we have found the phenomenon of α-rhythm “transfer” (translocation) (Figure 7) between bilateral symmetrical and anterio-posterior cortical zones to increase significantly under intense mental and physical work load, corresponding both to CAP type reconfigurations and functional displacements within a given CAP type. Thus, the intensity of brain activity and work dominants is expressed not only in the magnitude of activation reaction (coupled α-rhythm desynchronization and β- amplification, COD), but also in the speed of cortical EEG “mosaics” transformation. Fast FMA translocations and CAP reconfigurations are found in all experimental settings and psychological states, and prove to be significantly more expressed between bilateral symmetrical zones (switching dominant FMA every 1–2 s and faster) than in the anterio-posterior direction, although these parameters show marked individual specificity. Structural processing of EEG data shows the duration of structurally homogenous intervals not to exceed 0.25 seconds in a highly active cortex. On the other hand, on the background of relaxation and generalized α-rhythm amplification, the index of EEG stationarity can be seen to increase together with the decay interval of its autocorrelation function [40].

For selected EEG epochs, spectral analysis of β- and α-rhythm (K_β/α_) distribution needs to be carried out for all cortical zones from the same time-frame. Superposition of all obtained graphs (cf. Figure 6) enables to objectively analyze spectral characteristics of the EEG in various zones and to delineate FMAs.

The K_β/α_ coefficient can be expressed quantitatively in the following formula:

|  | (1) |
| --- | --- |

where *A_α_* and *A_β_* depict oscillation amplitudes corresponding to α- and β-frequencies, respectively; and where *T_B_* denotes time-periods of analysis.


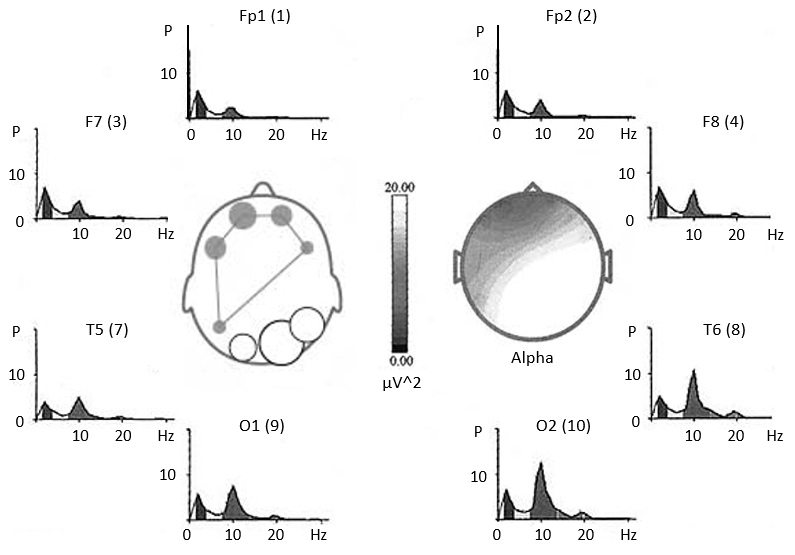


| Electrode | Delta | | Theta | | Alpha | | Beta1 | | Beta2 |
| --- | --- | --- | --- | --- | --- | --- | --- | --- | --- |
|  | P µV^2^ | Hz | P µV^2^ | Hz | P µV^2^ | Hz | P µV^2^ | Hz | P µV^2^ |
| Fp1 (1)  Fp2 (2)  F7 (3)  F8 (4)  T5 (7)  T6 (8)  O1 (9)  O2 (10) | 9.97  10.04  10.95  10.95  6.25  8.13  9.05  10.61 | 2.00  2.00  2.00  2.00  2.00  2.00  2.00  2.00 | 4.56  5.53  5.21  6.19  4.17  4.98  4.83  5.56 | 4.00  4.00  4.00  4.00  4.00  4.00  4.00  4.00 | 8.99  11.69  12.03  16.37  15.31  28.77  22.64  37.15 | 10.00  10.00  10.00  10.00  10.00  10.00  10.00  10.00 | 1.38  1.74  1.70  2.46  2.05  4.71  2.66  5.53 | 19.00  19.00  19.00  19.00  14.00  14.00  19.00  14.00 | 1.14  1.92  1.38  2.78  1.39  3.46  2.02  3.09 |

**Figure 6.** Example of EEG spectral analysis (in 0.5 s periods). The table above displays oscillation power (P, in µV) across frequency bands and recording sites. The graphs depict oscillation frequency (Hz, abscissa axis), and power (P, ordinate axis). Projected circles on the cortical hemispheres (above, left) show temporarily dominant regions with heightened activation (black circes: β-rhythms), and areas falling under coupled inhibition (white circles: α-rhythm). The size of black and white circles reflects relative regional activation and inhibition magnitude, respectively. Electrode localization is designated both by the 10–20 system, as well as our own schema (numbers in brackets, Table above) (Appendix 3).


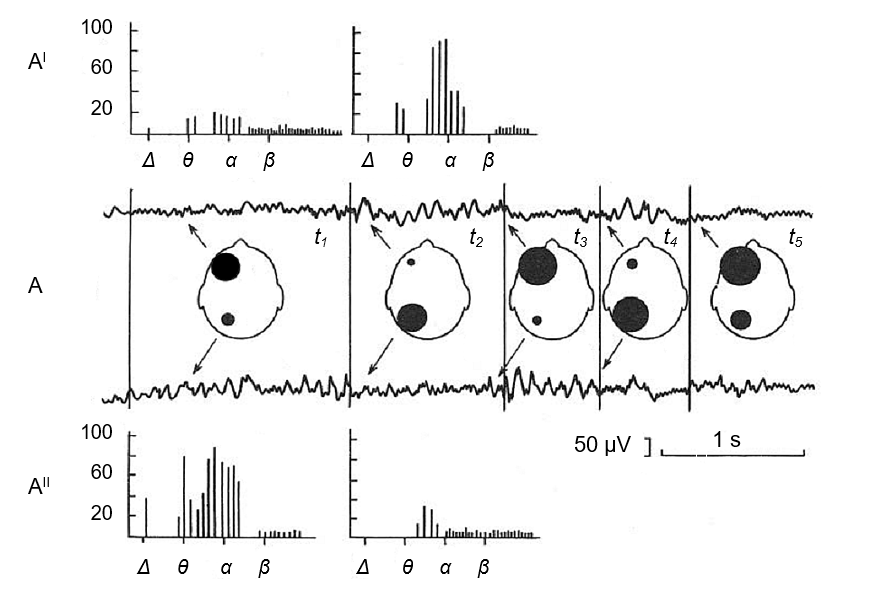


**Figure 7.** Graphic analysis of the spectral characteristics of structurally uniform EEG intervals **(**periods *t_1_*–*t_5_***).** A—examples of EEG segments from frontal and temporo-occipital regions of the left hemisphere; A^I^ and A^II^—spectral density graphs corresponding to the same recordings. Abscissa—oscillation frequency (Hz) of EEG waves, ordinate—their amplitude (µV). Black circle size on hemispheric projections reflects relative regional activation magnitude.

**Appendix 2**

The Eyes open/Eyes closed (EO/EC) test, described already by Hans Berger (1929), provides a simple and reliable means to assess quantitative differences in regional cortical activation [74]. It allows for functional testing of human brain states during highly engaged activity (such as oral exams), taking place on the background of highly desynchronized EEG and increased biological noise from various sources, such as interference from muscle potentials, corneoretinal potentials while blinking, *etc* [6], which drastically complicate background EEG analysis [75]. Experiments have shown that measures based on “activation reaction” in the EO/EC test convey identical information on the localization of maximal activation foci (FMA) as that given by spectral analysis of the K_β/α_ index [6].

Relaxational features of the β-rhythm (by EC) and the α-rhythm (by EO), *i.e.* the resetting speed of stationary regimes in oscillatory activity, are used to characterize excitation and inhibition inertia, and testify to the intra-cortical nature of these processes. This conclusion is based on comparing parameters of biorhythm frequency with the speed of synchronization and desynchronization reaction on the EC/EO test (Figure 8). In addition to latent reaction period (LRP) indices for EC/EO, we’ve employed the following activation coefficient:

| $K_{C/O }= \frac{LRP (EC)}{LRP (EO)}$ | (2) |
| --- | --- |

where K_C/O_ > 1 is an active state within the range 1 < K_C/O_ < 300; and where K_C/O_ < 1 is an inhibitory state within the range 1> K_C/O_ > 0.001.

In Figure 2, the values for K_C/O_ are calculated by the normalized formula:

| $K_{C/O }= \frac{LRP (EC)-LRP (EO) +0.1}{LRP (EC)+LRP (EO)}$ | (3) |
| --- | --- |

where values within K_C/O_ > 0 reflect activation; and values within K_C/O_ < 0 reflect inhibition.


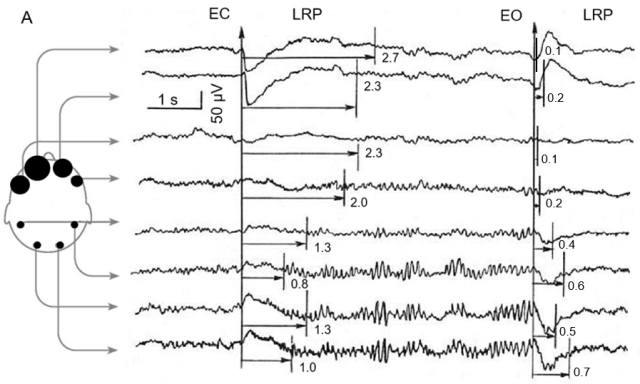

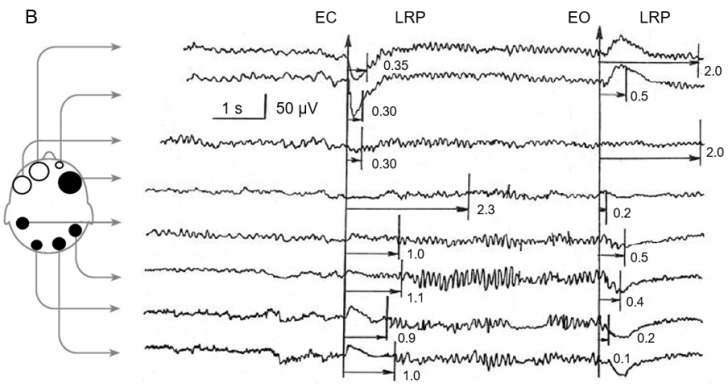


**Figure 8.** Two characteristic types of cortical activation patterns. A—executive function and logical effort (sequential analysis), dominance of frontal left brain regions; B—formation of images (simultaneous synthesis), dominance of posterior right gnostic regions**.** Levels of regional activation are calculated by LRP for EC/EO, the values of which are indicated by horizontal arrows and numbers (baseline 0 marks the appearance of corneoretinal potential by EC/EO). The size of black circles on hemispheric projections reflects relative regional activation magnitude; white circle size—relative inhibition magnitude.

Methods to determine ongoing cortical FMAs by using the EC/EO test, and methods to determine the extent and sign of functional asymmetry have independent significance. When comparing levels of cortical activation, functional asymmetry can be defined either by variational distrubution of K_C/O_ curves, or by using alternative methods, such as calculating the number of cases with significantly higher left- or right-sided K_C/O_ (at paired electrodes or inter-hemispherically). This has led to two groups of systemic EEG coefficients, characterizing cortical non-equilibria along anterio-posterior (fronto-occipital) (Eq 4) and inter-hemispheric (bilateral) (Eq 5) activation gradients.

| $\begin{matrix} K_{A/P}= \frac{anterior\Sigma K_{C/O}-posterior\Sigma K_{C/O}}{anterior\Sigma K_{C/O}+posterior\Sigma K_{C/O}} \\ \end{matrix}$ | (4) |
| --- | --- |

where A/P denotes anterio-posterior asymmetry,

| $\begin{matrix} K_{L/R}= \frac{left\Sigma K_{C/O}-right\Sigma K_{C/O}}{left\Sigma K_{C/O}+right\Sigma K_{C/O}} \\ \end{matrix}$ | (5) |
| --- | --- |

where L/R denotes bilateral asymmetry.

The coefficients K (A/P) and K (L/R) were also calculated by the parameter K_β/α_.

**Appendix 3**

In our EEG recordings we have generally not used the standard 10–20 system, but have preferred the R. U. Krönlein schema [76] to secure most accurate electrode placement on the speech zones of the left hemisphere. The method of symmetrical electrode placement developed by us on the basis of this schema [17,40] allows, by taking into consideration individual specifics of skull structure, to localize the speech zones of Broca and Wernicke with maximum accuracy, and thus to study their specific roles in regulating human activity.

The Krönlein schema is applied in the following manner (Figure 9, right). First, the lower horizontal (1) is determined, which passes through the lower margin of the eye socket and the upper margin of the external auditory canal. In parallel, an upper horizontal line (2) is drawn through the upper margin of the eye socket. Perpendicularly to these two horizontal lines are placed two vertical lines—the anterior vertical passes through the middle of the zygomatic arch, while the posterior vertical passes through the most posterior point at the base of the mastoid process. The projection of the central (Rolandic) fissure upon the skull is obtained by connecting two points: the first one corresponds to the intersection of the posterior vertical and the sagittal line, and corresponds to the apical end of the central fissure (Linea Rolandica). The second point is formed by the intersection of the anterior vertical and the upper horizontal (2), and corresponds to the lower end of the central fissure. The Linea Sylvii, which enables to localize the speech zones, is obtained by half-sectioning the angle between the Linea Rolandica and the upper horizontal, and extending the dividing midline till it intersects with the posterior vertical. The localization of Brodmann areas is taken into account in this schema (Figure 9, left).


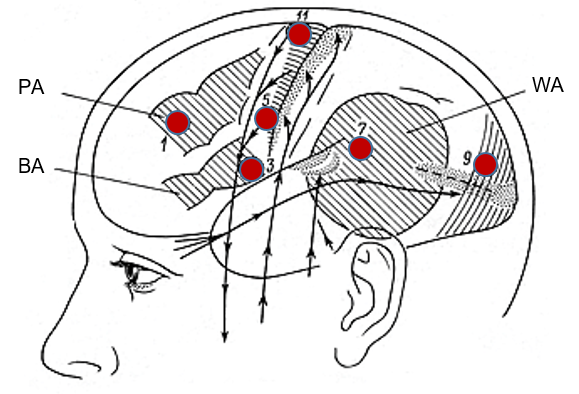

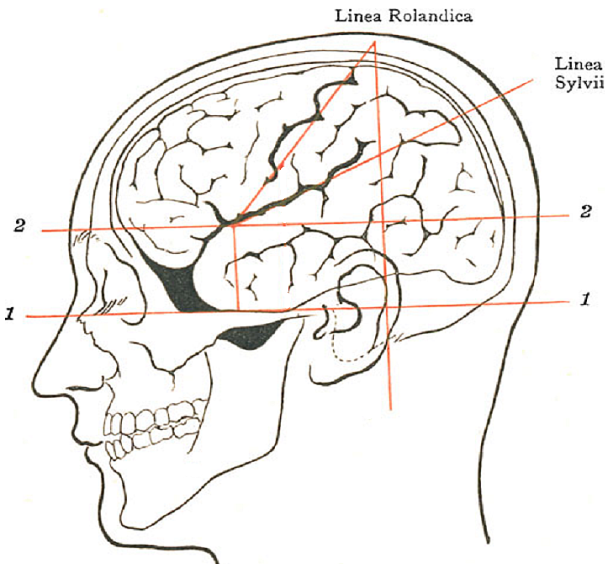


**Figure 9.** EEG recording sites and electrode montage. Left: Electrode placement on the left hemisphere. 1—prefrontal area (PA, Broadman area 46); 3—Broca’s area (BA, Broadman area 44); 5—hand area; 7—Wernicke’s area (WA, areas 39–40); 9—occipital area (visual fields 18–19). On the right hemisphere, electrodes 2, 4, 6, 8, and 10 are positioned symmetrically. Recording methods: unipolar, and with reference to the vertical top electrode, 11 (placed in front of the apical end of Rolandic fissure, on the interhemispheric fissure). Right: Krönlein’s elecrode montage schema [6,40,76]. О—lower horizontal; P—upper horizontal; R—apical end of Rolandic fissure; B (1)—anterior vertical; B (2)—posterior vertical.
